# Assessment of Automated Disease Detection in Diabetic Retinopathy Screening Using Two-Field Photography

**DOI:** 10.1371/journal.pone.0027524

**Published:** 2011-12-08

**Authors:** Keith Goatman, Amanda Charnley, Laura Webster, Stephen Nussey

**Affiliations:** 1 School of Medicine and Dentistry, University of Aberdeen, Aberdeen, Scotland; 2 St. George's Hospital, London, United Kingdom; National Institutes of Health, United States of America

## Abstract

**Aim:**

To assess the performance of automated disease detection in diabetic retinopathy screening using two field mydriatic photography.

**Methods:**

Images from 8,271 sequential patient screening episodes from a South London diabetic retinopathy screening service were processed by the Medalytix *iGrading*™ automated grading system. For each screening episode macular-centred and disc-centred images of both eyes were acquired and independently graded according to the English national grading scheme. Where discrepancies were found between the automated result and original manual grade, internal and external arbitration was used to determine the final study grades. Two versions of the software were used: one that detected microaneurysms alone, and one that detected blot haemorrhages and exudates in addition to microaneurysms. Results for each version were calculated once using both fields and once using the macula-centred field alone.

**Results:**

Of the 8,271 episodes, 346 (4.2%) were considered unassessable. Referable disease was detected in 587 episodes (7.1%). The sensitivity of the automated system for detecting unassessable images ranged from 97.4% to 99.1% depending on configuration. The sensitivity of the automated system for referable episodes ranged from 98.3% to 99.3%. All the episodes that included proliferative or pre-proliferative retinopathy were detected by the automated system regardless of configuration (192/192, 95% confidence interval 98.0% to 100%). If implemented as the first step in grading, the automated system would have reduced the manual grading effort by between 2,183 and 3,147 patient episodes (26.4% to 38.1%).

**Conclusion:**

Automated grading can safely reduce the workload of manual grading using two field, mydriatic photography in a routine screening service.

## Introduction

Diabetic retinopathy is one of the most common causes of vision loss in the developed world. Approximately three million people are thought to have diabetes in England. Since timely treatment is effective in reducing vision loss[1], everyone known to have diabetes and aged over twelve is invited for annual retinal screening using digital photography.

Screening generates a large amount of image data requiring grading. As the prevalence of diabetes continues to rise, there is a concomitant increase in the grading burden[2]. Furthermore, as in other screening programmes, the majority of images are normal. Automated grading has been proposed as a method to remove normal images from the manual grading queue, so reducing the overall manual workload.

A fully automated system using macular-centred images has been tested on a number of datasets, one of which included 33,535 consecutive patient episodes[3]–[5]. These studies were based on a one-field, staged mydriasis photographic protocol, hence confirmation was required that the results were applicable to other photographic protocols, such as two-field, mydriatic photography.

We report here the results of a study testing automated disease detection including quality assessment using images acquired and graded using two-field, mydriatic photography.

## Methods

### Data collection

Images from 8,500 consecutive patient episodes (approximately 6 months of screening data) were extracted from the Wandsworth, Richmond and Twickenham diabetic retinopathy screening service (DRSS), South London in 2009. Duplicate visits, non-screening follow-up visits and test images were excluded, leaving 8,271 unique patient episodes and 36,236 retinal photographs. The retinal photographs were nominally 45 degree fields of view acquired using a non-mydriatic fundus camera: 68% were from Topcon NW6 fundus cameras with Nikon D80 digital single lens reflex camera (DSLR) backs, while the remainder were acquired using Canon CR6 non-mydriatic fundus cameras with a Canon D30, 30D or 40D DSLR back. The resulting colour images were between 3.1 and 10.1 megapixels and were stored using high quality JPEG compression. All patients underwent routine mydriasis with Tropicamide 1% and, where present, both eyes were imaged. Two fields were taken per eye: a macula-centred and a disc-centred view. Where appropriate, more than one image of each field was taken at the discretion of the retinal screener.

### Manual Image grading

Images from all the patient episodes were routinely graded by the local DRSS, independently of this study, following the standard grading Pathway 2 (“full disease grading”) of the English National Screening Programme for diabetic retinopathy[6]. Where the photographic quality was deemed adequate, the eye was graded for both retinopathy and maculopathy, as per Table 1. The grade for each eye was a combined assessment of the macular and disc fields. Where multiple images were taken of a field, these were all included in the assessment; the grade for the eye was taken as the worst grade of all the fields acquired for the eye. The grade for the patient episode was taken from the eye with the more serious disease. Disagreements between the DRSS grade and the automated result were sent for internal arbitration. Disagreements pertaining to an episode originally graded as referable by the DRSS were also sent for external arbitration.

**Table 1:** Retinopathy and maculopathy grading scheme for the English National Screening Programme for Diabetic Retinopathy.

Retinopathy (R)		
R0	None	No visible retinopathy
R1	Background	Any microaneurym, haemorrhage or exudates
R2	Pre-proliferative	Venous beading, loop or reduplication Intraretinal microvascular abnormality (IRMA)Multiple deep, round or blot haemorrhages
R3	Proliferative	New vessels on the disc (NVD)New vessels elsewhere (NVE)Pre-retinal or vitreous haemorrhagePre-retinal fibrosis (with or without detachment)

Maculopathy (M)		
M0	None	No visible maculopathy
M1	Maculopathy	Exudate within 1 disc-diameter (DD) of the foveaExudate circinate or group within 1DD of foveaMicroaneurysm or haemorrhage within 1DD of fovea if visual acuity is poorer than 6/12.

Image quality	
Macular-centred field	The macula-centred field is considered adequate if both:• The centre of the fovea is at least 2DD from the edge of the field of view• Vessels are visible with 1DD of the centre of the fovea.
Disc-centred field	The disc-centred field is considered adequate if both:• The complete disc is at least 2DD from the edge of the field of view.• Fine vessels are visible on the disc.

### Automated image analysis

Automated grading software was purchased for this project (Medalytix *iGrading*™ system, 7 Water Street, Liverpool, L2 0RD) and run on a HP Proliant DL380 server at St George's Hospital. The software is designed to remove normal images from the manual grading queue. It does this by first checking the image for adequate clarity and field of view[3] before looking for the early signs of retinopathy. It may be operated in two modes: the first looks only for microaneurysms (MA)[3], while the second (MA/BH/EX) also looks for blots and exudates in addition to microaneurysms[4]. The software was evaluated here using both fields from each eye, and also using only the macula-centred field from each eye. Hence there were four automated strategies tested: MAs only on macular field alone, MA/BH/EX on macular field alone, MAs on the macular and disc fields, and MA/BH/EX on the macular and disc fields. As for manual grading, where more than one image of a field was acquired these were all included in the automated analysis: if any of the images were positive then that eye was treated as positive.

### Internal arbitration of disease/no-disease discrepancies

Images where there was a discrepancy between the manual and automated systems regarding whether disease was present were internally arbitrated by one of two “arbitration level” graders within the screening programme (SN and AC). Occasionally images were re-graded jointly. The arbitration outcomes were simply disease or no-disease. Since full grading was not performed the severity of any disease missed by the original DRSS grading could not be categorized.

### External arbitration of referable/non-referable discrepancies

Where there was a discrepancy between the DRSS grade and an automated strategy regarding whether an episode was referable these images were re-graded externally. Graders working in different screening programmes in England were invited to participate at the British Association of Retinal Screeners annual meeting in 2010. The grading took place in February and March 2011, using a web-based grading system based on the features of the English grading scheme. Graders were able to practise and become familiar with the system prior to carrying out the arbitration grading. A similar number of control images, where the manual and automated system agreed on the presence or absence of retinopathy, were added so that the graders did not know whether they were grading a discrepancy. Graders were able to view all the available images for each eye and each grader was shown the eyes in a random order. Twenty-five graders from 16 different English screening programmes took part. Of these 11 were arbitration-level graders, 8 secondary-level graders and 6 primary-level graders.

A consensus criterion, based on the grading of the eleven arbitration level graders, was used to determine whether each eye was referable. An eye was considered referable if at least 8/11 of the arbitration-level graders indicated referable features, and non-referable when 3/11 or fewer of the arbitration-level graders indicated a referable feature. When between 4 and 7 graders indicated a referable feature it was deemed there was insufficient consensus to assign a grade.

### Statistical comparison

The sensitivities of the four automated strategies were compared for the detection of any retinopathy, referable retinopathy, unassessable episodes, and for each grade of retinopathy listed in Table 1. The workload reduction was calculated as the proportion of images the automated system recorded as both gradeable and without disease. 95% confidence intervals were calculated on all measurements.

### Information Governance

All the images were abstracted without patient identifiers or grading information. Manual grading was done prior to image analysis and without knowledge of the study. Arbitration grading was done without knowledge of either the software analysis or prior grading results. Only one person (LW), who was not involved in any grading or software analysis, had access to both grading results and image analyses. The study had written consent from the Chairman of the London Wandsworth Research Ethics Committee and the Institutional Caldicott Guardian. All participants in the English National Screening Programme for Diabetic Retinopathy agree to the anonymous use of their images for teaching and research. The study had no effect on the routine clinical care of patients within the screening programme. Where there was any question of the effect of grading on patient management the electronic records were accessed and the subsequent outcome examined.

## Results

### Manual grading

Episodes in which the manual and automated systems disagreed were passed to internal arbitration grading. If the disagreement involved a referable episode then these were also passed to external arbitration grading. Of the 8,271 patient episodes included in the study, four were removed following external arbitration as no consensus was reached regarding their grade, leaving 8,267 episodes. Of these, 4.2% were considered unassessable by manual grading. 58.7% were graded as having no retinopathy, 30.0% were graded as having background retinopathy (R1), 4.8% were graded as having maculopathy (M1), 1.6% were graded as having pre-proliferative retinopathy (R2), and 0.7% were graded as having proliferative retinopathy (R3). Overall, 587 (7.1%) were given referable grades (i.e. R2, R3 or M1).

### Internal arbitration of disease/no disease discrepancies

Of the disease/no-disease discrepancies there were 48 episodes where the original DRSS grade was disease (R1 or above) but the arbitration grade was R0 (no disease). These downgraded discrepancies included eleven referable episodes: one originally graded R3 (proliferative retinopathy) and 10 graded as M1 (maculopathy). All of these episodes were included in the external arbitration. There were 326 episodes whose original DRSS grade was R0 (no disease) but arbitration found disease. Since full grading was not performed the disease missed by the DRSS grading could range from background (R1) to proliferative retinopathy (R3). The significant increase in the number of images categorised as having disease following internal arbitration suggests the arbitration process operated at a higher disease sensitivity than the routine screening service.

### External arbitration of referable/non-referable discrepancies

Referable/non-referable discrepancies from any of the four automated grading strategies were arbitrated externally. All of discrepancies concerned maculopathy (R1M1), except for a case where the original DRSS grade was proliferative retinopathy (R3) but internal arbitration had downgraded it to no disease (R0). Combining the discrepancies from the four automated strategies resulted in 32 eyes for external arbitration. A further 48 eyes, in which the manual and automated grading agreed, were also included as control images. The median time for all levels of grader to complete the 80 eyes was 2.6 hours (interquartile range 2.0 to 3.4 hours).

**Figure 1:**
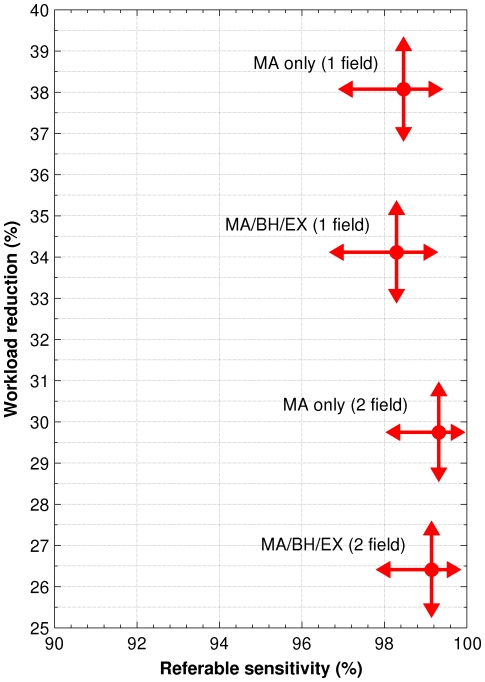
Receiver operator characteristic (ROC) plot for all the graders who took part in the external arbitration. The three levels of grader (primary, secondary and arbitration) are shown by ‘*’, ‘+’ and ‘o’ respectively. All the graders were compared against a consensus grading calculated from the results from all the arbitration level graders. Notice even amongst the arbitration level graders there is a range of operating points from high specificity and lower sensitivity to high sensitivity and lower specificity.

Figure 1 shows a receiver operator characteristic (ROC) plot for all the graders who completed the external arbitration. The three levels of grader (primary, secondary and arbitration) are indicated separately, since primary level graders may be expected to have a different sensitivity/specificity trade-off to arbitration level graders. Nevertheless, even amongst arbitration level graders there is a range of operating points from high specificity and lower sensitivity to high sensitivity and lower specificity.

Of the 32 discrepancies presented to the graders, 28/32 achieved a consensus grade; the remaining 4 eyes were excluded from the analysis. Two of the episodes were considered unassessable by the arbitrators and nine were graded as non-referable (including the episode originally DRSS graded as having proliferative retinopathy which was internally arbitrated as R0). The remaining 17 eyes were graded as having maculopathy. However, three of these had only red lesions within the macula and normal visual acuity and so would have been graded as background retinopathy (R1) according to the English National Screening Programme for Diabetic Retinopathy grading scheme.

Hence, of the original 32 discrepancies, 2 unassessable eyes and 14 eyes with maculopathy were missed by the automated system. Of these, only 6/16 were unanimously graded as unassessable or referable by the arbitration level graders. Including the grading from the primary and secondary graders there were only 3/16 images which were unanimously graded as unassessable or referable by graders of all levels.

### Automated grading

Table 2 lists the sensitivities for automated disease detection for each grade, as well as for any diabetic retinopathy and any referable diabetic retinopathy. The associated workload reduction of employing the automated strategy as a disease/no-disease grader is also shown. The sensitivity for detecting unassessable images ranged from 97.4% (MA only, macular field) to 99.1% (MA/BH/EX, both fields). The sensitivity for detecting any retinopathy ranged from 89.9% (MA/BH/EX, macular field) to 95.8% (MA only, both fields). The sensitivity for referable retinopathy (i.e. M1, R2 and R3) ranged from 98.3% (MA/BH/EX, single field) to 99.3% (MA only, both fields). The workload reduction ranged from 26.4% (MA/BH/EX, both fields) to 38.1% (MA only, macular field). The sensitivities for pre-proliferative and proliferative disease were 100% using all four automated strategies.

**Table 2:** Performance of the four automated strategies for detecting different grades of disease, together with the associated workload reduction.

DRSS category	Number identified by the automated system	Proportion identified (%) [95% confidence interval]
**MA/BH/EX (both fields)**		
**Unassessable**	343/346	99.1 [97.5 to 99.7]
**R0**	3094/5141	60.2 [58.8 to 61.5]
**R1**	2065/2193	94.2 [93.1 to 95.1]
**M1**	390/395	98.7 [97.1 to 99.5]
**R2**	131/131	100 [97.2 to 100]
**R3**	61/61	100 [94.1 to 100]
**Any retinopathy**	2647/2780	95.2 [94.4 to 95.9]
**Referable**	582/587	99.1 [98.0 to 99.6]
**(Workload reduction**	**2183/8267**	**26.4 [25.5 to 27.4])**
		
**MA(both fields)**		
**Unassessable**	339/346	98.0 [95.9 to 99.0]
**R0**	2806/5141	54.6 [53.2 to 55.9]
**R1**	2080/2193	94.8 [93.8 to 95.7]
**M1**	391/395	99.0 [97.4 to 99.6]
**R2**	131/131	100.0 [97.2 to 100.0]
**R3**	61/61	100 [94.1 to 100]
**Any retinopathy**	2663/2780	95.8 [95.0 to 96.5]
**Referable**	583/587	99.3 [98.3 to 99.7]
**(Workload reduction**	**2459/8267**	**29.7 [28.8 to 30.7])**
		
**MA/BH/EX (macular field only)**		
**Unassessable**	342/346	98.8 [97.1 to 99.5]
**R0**	2607/5141	50.7 [49.3 to 52.1]
**R1**	1921/2193	87.6 [86.2 to 88.9]
**M1**	385/395	97.5 [95.4 to 98.6]
**R2**	131/131	100.0 [97.2 to 100.0]
**R3**	61/61	100 [94.1 to 100]
**Any retinopathy**	2498/2780	89.9 [88.7 to 90.9]
**Referable**	577/587	98.3 [96.9 to 99.1]
**(Workload reduction**	**2820/8267**	**34.1 [33.1 to 35.1])**
		
**MA(macular field only)**		
**Unassessable**	337/346	97.4 [95.1 to 98.6]
**R0**	2229/5141	43.4 [42.0 to 44.7]
**R1**	1976/2193	90.1 [88.8 to 91.3]
**M1**	386/395	97.7 [95.7 to 98.8]
**R2**	131/131	100 [97.2 to 100]
**R3**	61/61	100 [94.1 to 100]
**Any retinopathy**	2554/2780	91.9 [90.8 to 92.8]
**Referable**	578/5877	98.5 [97.1 to 99.2]
**(Workload reduction**	**3147/8267**	**38.1 [37.0 to 39.1])**

**Figure 2:**
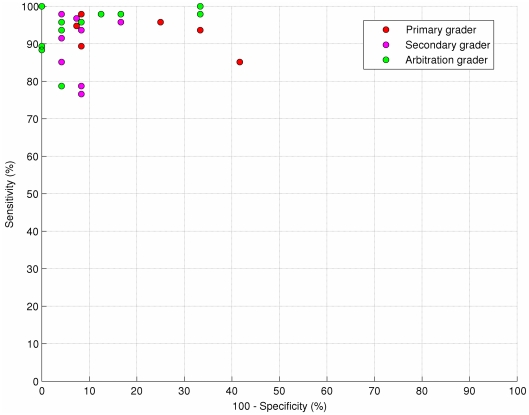
Workload reduction versus referable sensitivity for the four automated strategies. The arrows indicate the 95% confidence intervals on the measurements.

Figure 2 shows a plot of workload reduction versus referable disease sensitivity using the four automated strategies. The arrows indicate the 95% confidence intervals on the measurements. While including the disk centred field detected additional referable cases, it also resulted in many more non-referable cases being detected, which would require manual grading. For both the MA only and MA/BH/EX strategies, including the disk centred field picked up five additional referable maculopathy cases not detected using the macular field alone. However, in order to find these five additional cases an additional 688 non-referable cases were found using MAs only, or an additional 637 cases using MA/BH/EX. Hence, of the additional cases found by including the disk centred field, only 5/688 (0.73% [0.31% to 1.69%]) were referable using MAs only, and 5/637 (0.78% [0.34% to 1.82%]) were referable using MA/BH/EX.

## Discussion

There are three previous published reports of the performance of this automated system [3]–[5] using staged mydriasis and single-field photography. The present study assessed the performance of automated disease detection on an unselected population of 8,271 subjects from a South London retinal screening service using mydriatic, two-field photography. The sensitivities for referable disease using the four automated strategies are very similar to those published previously with the same techniques using a macular centred, mydriatic photographic protocol (Table 3).

**Table 3:** Comparison of per patient sensitivities and workload reduction from this study and three Scottish studies using the same software.

	MA (MAs only)				MA/BH/EX (MAs + blots + exudates)			
Study	Unassessable (%)	Referable (%)	R2/R3 (%)	Workload reduction (%)	Unassessable (%)	Referable (%)	R2/R3 (%)	Workload reduction (%)
**This study** Macula field only	97.4 [95.1,98.6](337/346)	98.5[97.1,99.2](578/587)	100[98.0,100](192/192)	38.1[37.0,39.1](3147/8267)	98.8[97.1,99.5](342/346)	98.3[96.9,99.1](577/587)	100[98.0,100](192/192)	34.1[33.1,35.1](2820/8267)
**This study** Both fields	98.0[95.9,99.0](339/346)	99.3[98.3,99.7](583/587)	100[98.0,100](192/192)	29.7[28.8,30.7](2459/8267)	99.1[97.5,99.7](343/346)	99.1[98.0,99.6](582/587)	100[98.0,100](192/192)	26.4[25.5,27.4](2183/8267)
**Philip et al. 2007** [3]	99.8[99.0–100](552/553)	98.0[95.3,99.1](241/246)	100[94.6,100](67/67)	45.7[44.5,46.9](3070/6722)				
**Fleming et al. 2010a** [4]	98.6[97.4,99.3](634/643)	95.0[93.5,96.1](1076/1133)	97.6[95.9,98.6](488/500)	39.2[38.1,40.3](2971/7586)	98.8[97.6,99.4](635/643)	96.9[95.7,97.8](1098/1133)	98.2[96.6,99.1](491/500)	38.9[37.8,40.0](2951/7586)
**Fleming et al. 2010b** [5]	99.8[99.5,99.9](1824/1827)	98.1[97.3,98.7](1603/1634)	100[99.2,100](504/504)	38.4[37.8,38.9](12154/31681)				

Note that study [4] is the only one that apparently missed proliferative disease. However, subsequent re-grading of the six supposed proliferative cases downgraded all cases to non-referable (the six images in question are available as supplementary material from the BJO website).

The performance of automated grading should be compared with that achieved by manual grading of photographic images. Several studies have demonstrated the limited sensitivity and specificity of retinal photography for detecting referable retinopathy: some referable disease is missed before the eye is even graded[7]–[9]. Furthermore, image grading is not a trivial task and other studies have reported wide disagreement between graders[10]–[13]. The ROC plot in figure 1 indicates the range of performance that may be expected from different levels of grader within a screening service. Images are often difficult to interpret owing to subtle and inconclusive disease features, the presence of distracting artefacts or the borderline position of lesions. Finally, very occasionally, a grader makes a mistake and misses a clear feature. In the external arbitration exercise only 16 of the 32 eyes originally DRSS graded as referable, but graded negative by one or more of the automated strategies, were graded as referable by a consensus of the arbitration level graders. Of these only six were unanimously graded as referable, meaning that at least one arbitration level grader would also have missed each of the remaining 10 cases.

Clinical follow-up information was available for 11/14 episodes that were graded as normal by the automated system but as referable maculopathy by external arbitration. Of these, all but three went for optical coherence tomography (OCT). One of the patients had laser treatment 10 months after screening. Other studies have shown that maculopathy and macular oedema progresses slowly [14], [15].

There was a significant difference in workload reduction depending on whether the macular field alone or both fields were used (Figure 1). Automated grading using microaneurysms alone achieved a workload reduction of 38.1% (CI 37.0% – 39.1%) with the macular field alone and 29.7% (CI 28.8% – 30.7%) with both fields. The addition of the disc-centred field may be expected to increase sensitivity, since the manual and automated systems both have a second opportunity to spot retinopathy. However, it is also an additional opportunity to detect false positive disease which decreases the workload reduction. In this study less than 0.8% of the additional cases generated by including the disk centred field were referable. No advantage was found using MA/BH/EX over MAs alone. MA/BH/EX resulted in similar sensitivity but at a higher false positive rate that decreased the associated workload reduction.

No attempt has been made to estimate the cost-benefits associated with the workload reduction, as this will depend on the staffing numbers, salary grades and work throughput in individual screening services. However, it may be expected that a 38% workload reduction applied to a screening programme would have considerable benefits, especially given the increasing prevalence of diabetes.

In conclusion, an automated disease/no-disease grading system has been tested against data from a retinal screening service using a mydriatic two-field, photographic protocol. Automated grading can safely reduce the burden of manual grading with potential cost-benefits.
